# Physiological Chemistry

**Published:** 1856-01

**Authors:** 


*Physiological Chemistry:* by Prof. C. G. LEHMANN. Translated from the second edition by GEORGE E. DAY, M. D., F. R. S., &c., &c. Edited by R. E. ROGERS, M. D., Prof. of Chemistry in the Medical Department of the University of Pennsylvania, with illustrations selected from Funke's Atlas of Physiological Chemistry, and an appendix of Plates. Complete in two volumes. Philadelphia: Blanchard & Lea, 1855.

This translation appeared in England, under the auspices of the "Cavendish Society." This alone is a guaranty of the value of the work. The author, however, is already well known to the American professional public. His contributions to physiology, are incorporated into all our text-books. We had intended to give our readers a synopsis of some of the more important portions of the work, but we cannot find room, and we yield to this necessity the more cheerfully from the conviction which we feel, that every one interested in physiological science, will possess the work for himself.

For sale by D. B. Cooke & Co. J.

